# MicroRNA‐383 inhibits doxorubicin resistance in hepatocellular carcinoma by targeting eukaryotic translation initiation factor 5A2

**DOI:** 10.1111/jcmm.14197

**Published:** 2019-02-23

**Authors:** Chaoyong Tu, Wei Chen, Shuqian Wang, Wei Tan, Jingqiang Guo, Chuxiao Shao, Weilin Wang

**Affiliations:** ^1^ Department of Hepatobiliary and Pancreatic Surgery The Second Affiliated Hospital School of Medicine Zhejiang University Hangzhou Zhejiang P.R. China; ^2^ Department of Hepatobiliary and Pancreatic Surgery, Lishui Hospital Zhejiang University School of Medicine, The Fifth Affiliated Hospital of Wenzhou Medical University Lishui Zhejiang P.R. China; ^3^ Tongde Hospital of Zhejiang Province Cancer Institute of Integrated traditional Chinese and Western Medicine Zhejiang Academy of Traditional Chinese Medicine Hangzhou Zhejiang China; ^4^ Division of Breast Surgery, Department of Surgery The First Affiliated Hospital, School of Medicine, Zhejiang University Hangzhou Zhejiang P.R. China; ^5^ Key Laboratory of Precision Diagnosis and Treatment for Hepatobiliary and Pancreatic Tumor of Zhejiang Province The First Affiliated Hospital, School of Medicine, Zhejiang University Hangzhou Zhejiang P.R. China; ^6^ Clinical Research Center of Hepatobiliary and Pancreatic Diseases of Zhejiang Province, School of Medicine The First Affiliated Hospital, Zhejiang University Hangzhou Zhejiang P.R. China; ^7^ State Key Laboratory & Collaborative Innovation Center for Diagnosis and Treatment of Infectious Disease Zhejiang University Hangzhou Zhejiang P.R. China

**Keywords:** chemoresistance, EIF5A2, hepatocellular carcinoma, MiR‐383

## Abstract

Drug resistance occurs commonly in cancers, especially in hepatocellular carcinoma (HCC). Accumulating evidence has demonstrated that microRNAs (miRNAs) play a vital role in tumour chemoresistance. However, little is known about the role of miR‐383 in HCC chemoresistance. In the present study, RT‐PCR and western blotting were used to identify the expression profile of miR‐383 and eukaryotic translation initiation factor 5A2 (EIF5A2). The bioinformatics website Targetscan was used to predict the target genes of miR‐383. In vitro and in vivo loss‐ and gain‐of‐function studies were performed to reveal the effects and potential mechanism of the miR‐383/EIF5A2 axis in chemoresistance of HCC cells. The expression level of miR‐383 correlated negatively with doxorubicin (Dox) sensitivity. Overexpression of miR‐383 promoted HCC cells to undergo Dox‐induced cytotoxicity and apoptosis, whereas miR‐383 knockdown had the opposite effects. *EIF5A2* was predicted as a target gene of miR‐383. *EIF5A2* knockdown sensitized HCC cells to Dox. Moreover, miR‐383 inhibition‐mediated HCC Dox resistance could be reversed by silencing *EIF5A2*. Finally, we demonstrated that miR‐383 inhibition could enhance Dox sensitivity by targeting *EIF5A2* in vivo. The results indicated that miR‐383 inhibited Dox resistance in HCC cells by targeting *EIF5A2*. Targeting the miR‐383/EIF5A2 axis might help to alleviate the chemoresistance of HCC cells.

## 1 INTRODUCTION

Hepatocellular carcinoma (HCC) is one of the most frequent malignancies worldwide, and is the third leading cause of cancer‐related death.^1^ Surgical resection and liver transplantation are the main therapeutic strategies used to treat this disease; however, they are only suitable for patients with early‐ to mild‐stage HCC.^2^, ^3^ For patients with the most advanced HCC, transarterial chemoembolization (TACE) is the most frequently selected treatment.^4^ Doxorubicin (Dox) is a first‐line chemotherapy agent for TACE.^5^, ^6^, ^7^ However, drug resistance of HCC cells greatly limits the efficacy of Dox.^8^ Therefore, there is an urgent need to understand the molecular mechanisms involved in the Dox resistance of HCC and identify novel targets to alleviate chemoresistance and improve clinical outcome.

MicroRNAs (miRNAs) are endogenous, highly conserved small non‐coding RNAs that regulate gene expression at the post‐transcriptional level by binding to the 3′‐untranslated region (3′ UTR) of target mRNAs.^9^ Increasing evidence suggests that dysregulated miRNAs are involved in tumourigenesis, tumour progression and chemoresistance.^10^ The restoration or knockdown of several miRNAs has been proven to relieve or enhance drug resistance in HCC.^11^ However, there are large gaps in our understanding of the potential roles of miRNAs in HCC chemoresistance, and we lack efficient and specific miRNA targets to overcoming HCC chemoresistance. Therefore, it is essential to identify key candidate miRNAs that regulate HCC chemoresistance to improve treatment outcome.

MicroRNA‐383 acts as a tumour suppressor in many cancers, including oesophageal squamous cell carcinoma, glioma, lung cancer and others.^12^, ^13^, ^14^. Chen et  al reported that miR‐383 might inhibit HCC cell proliferation partially via down‐regulating *APRIL* (encoding a proliferation‐inducing ligand) expression.^15^ Fang et  al showed that miR‐383 is down‐regulated in HCC and acts as a tumour suppressor by targeting *LDHA* (encoding lactate dehydrogenase A).^16^ However, the role of miR‐383 in HCC chemoresistance remains unclear.

Thus, in the present study, we aimed to investigate the role miR‐383 in HCC chemoresistance and reveal its potential mechanism. We found that overexpression of miR‐383 could promote Dox sensitivity in HCC cells. Further study showed that *EIF5A2* (encoding eukaryotic translation initiation factor 5A2) is a target gene of miR‐383, and miR‐383 could sensitize HCC cells to Dox by regulating *EIF5A2* in vitro and in vivo.

## 2 MATERIALS AND METHODS

### 2.1 Cell culture

Human HCC cell lines (Huh‐7, HepG2, SUN‐387 and SUN‐449) were purchased from the Type Culture Collection of the Chinese Academy of Sciences (Shanghai, China). Cell lines Huh‐7 and HepG2 were routinely cultured in Dulbecco's modified Eagle's medium (DMEM) (Gibco, Rockville, MD) supplemented with 10% foetal bovine serum and 1% penicillin/streptomycin mix (Sigma‐Aldrich, St. Louis, MO) at 37°C and 5% CO_2_ in a humidified environment, and allowed to grow to confluence. Cell lines SUN‐387 and SUN‐449 were cultured in Roswell Park Memorial Institute (RPMI)‐1640 medium under the same conditions. Dox was purchased from Sigma‐Aldrich Co.

### 2.2 Real‐time PCR analysis

Total RNAs, including miRNAs, were extracted using the RNAiso reagent (Takara, Dalian, China), according to the manufacturer's instructions. For the quantitative detection of *EIF5A2* mRNA expression, PCR amplification was performed with SYBR Green PCR Master Mix (Takara). For the quantitative detection of miR‐383 expression, PCR amplification was carried out using a Mir‐X™ miRNA quantitative real‐time reverse transcription PCR (qRT‐PCR) TB Green™ Kit (Takara), based on the manufacturer’s protocols. *GAPDH* (encoding glyceraldehyde‐3‐phosphate dehydrogenase) was used as the internal reference for normalization of *EIF5A2 *mRNA expression. U6 was used as the internal reference for normalization of miR‐383 expression. Relative gene expression was calculated using the 2^−ΔΔCt^ method.^17^

### 2.3 Western blotting

Proteins were exacted from cells using radioimmunoprecipitation assay (RIPA) buffer containing protease inhibitors. The protein concentration was quantified using a bicinchoninic acid (BCA) Protein Assay Kit (Pierce, Rockford, IL). An equal amount of protein (40 µg) was separated by gel electrophoresis, and then transferred to a polyvinylidene fluoride membrane. The membrane was blocked with 5% non‐fat milk and subsequently incubated with anti‐EIF5A2 primary antibodies (dilution 1:1000; Abcam, Cambridge, UK) and anti‐GAPDH primary antibodies (Abcam) at 4°C overnight. The membrane was then probed with corresponding secondary antibodies (dilution 1:2000; Abcam) at room temperature for 1 hour. Immunoreactive protein bands were visualized using an ECL Substrate Kit (Abcam). Glyceraldehyde‐3‐phosphate dehydrogenase was used as loading control.

### 2.4 Cell viability assay

Cell viability was measured using a cell counting kit‐8 (CCK‐8; Dojindo, Kumamoto, Japan) assay. Briefly, cells were seeded into a 96‐well plate at 3 × 10^3^ cells/well and cultured overnight. Cells with different treatments were treated with various concentrations of Dox for 24 hours. The cells were then treated with 10 µL of CCK‐8 reagent and cultured at 37°C for 2 hours according to the manufacturer's protocol. The absorbance was determined at 450 nm using an MRX II microplate reader (Dynex, Chantilly, VA).

### 2.5 Cell transfection

The miR‐383 mimics, inhibitor and negative controls were purchased from GenePharma (Shanghai, China). The EIF5A2 short interfering RNA (siRNA) was also obtained from GenePharma. Cell transfection of miRNAs or EIF5A2 siRNAs was performed with Lipofectamine 2000 (Invitrogen, Carlsbad, CA) according to the manufacturer's instructions. The transfection medium was replaced with a complete medium 6 hours after transfection, and the cells were incubated for the indicated times. All treatments were started 24 hours after transfection.

### 2.6 Cell apoptosis assay

Cell apoptosis was determined using the Annexin V fluorescein isothiocyanate (FITC)/propidium iodide (PI) kit (Invitrogen). Briefly, the cells were plated into 6‐well plates at a density of 3 × 10^5^ cells/well, and then transfected with miR‐3383 mimics, inhibitors or controls and treated with 1 µg/mL of Dox. After 12 hours, the cells were collected and stained with Annexin V‐FITC and PI. Flow cytometry using a BD CANTO II instrument (BD, Franklin Lakes, NJ) was then performed to detect apoptosis of the transfected cells.

### 2.7 Animal model

Huh7 cells (5 × 10^6^ cells per mouse) were slowly injected into the right‐side flanks of male BALB/c nude mice aged 3‐5 weeks. After the tumour grew to 0.5 mm, the mice were divided into four groups of five mice each. The groups of mice received normal saline (50 µL), PBS containing Dox (3 mg/kg), agomiR‐383 (2 nmol per mouse in 50 µL) or PBS containing Dox and 2 nmol of agomiR‐383. PBS, Dox and agomiR‐383 were administered by tumour injection every 3 days. The volume of the tumour was monitored and calculated using the following formula: volume = (length × width^2^)/2. The mice were weighed every 3 days. The mice were killed 2 weeks later, and the tumours were extracted, weighed and frozen in liquid nitrogen or fixed in 10% buffered neutral formalin for further analysis. All animal procedures and experimental protocols were approved by the Laboratory Animal Ethics Committee of our hospital.

### 2.8 Immunohistochemistry

Immunohistochemical staining of the paraffin‐embedded sections from mouse tumour tissues was performed with a microwave‐based antigen retrieval technique, and specimen slides were incubated overnight at 4°C with primary antibodies raised against Ki67 (Cell Signaling Technology). The slides were then observed and photographed under a light microscopy (Olympus, Tokyo, Japan). The positive rates were measured using Image‐Pro Plus v. 6.0 software (Media Cybernetics, Bethesda, MD).

### 2.9 Terminal deoxynucleotidyl transferase dUTP nick end labelling assay

The terminal deoxynucleotidyl transferase dUTP nick end labelling (TUNEL) assay was used to identify apoptosis in 5‐mm sections of paraffin‐embedded nude mouse tissues with an in situ cell death detection kit obtained from Roche (Basel, Switzerland) according to the manufacturer's instructions. The apoptotic cells were observed under a light microscope (Olympus). Briefly, the TUNEL‐positive cells that showed green nuclear staining and all cells with blue nuclear DAPI (4',6‐diamidino‐2‐phenylindole) staining were counted within five randomly selected fields under high‐power magnification (DM‐2500; Leica Microsystems, Wetzlar, Germany). The index of apoptosis was expressed as the ratio of positively stained apoptotic cells to the total number of cells counted ×100%.

### 2.10 Statistical analysis

All assays were performed in triplicate. The data are expressed as the mean ± SD. The statistical analyses were performed with Student's *t* test. *P* < 0.05 was considered to indicate a statistically significant difference. Statistical analyses were performed with spss software, version 18.0 (IBM, Armonk, NY).

## 3 RESULTS

### 3.1 The expression level of miR‐383 was negatively associated with Dox sensitivity in HCC cells

**Figure 1:**
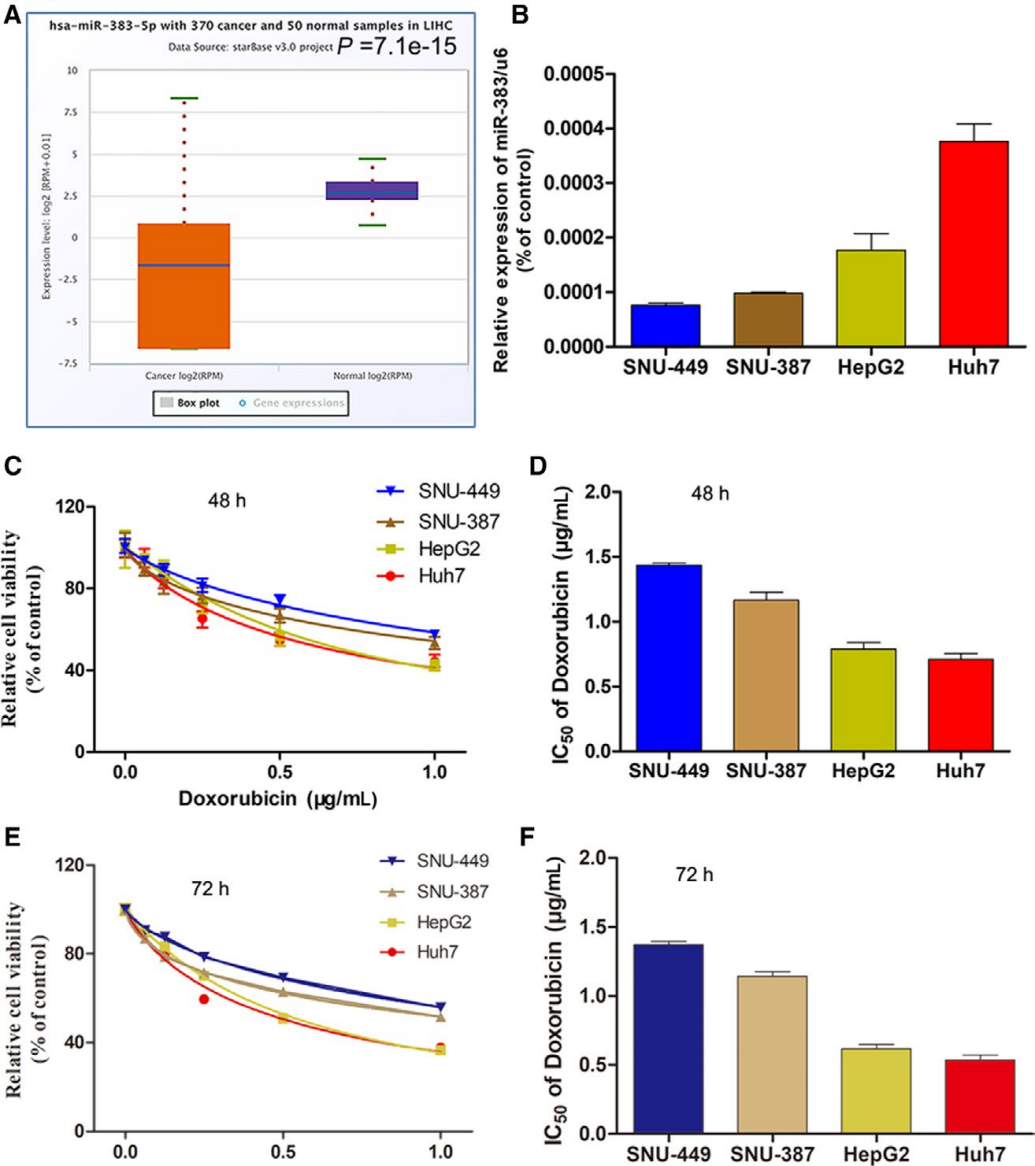
MicroRNA‐383 expression is negatively related to Dox sensitivity in HCC cells. A, We used starBase v 3.0 project to analyse the level of miR‐383 in 370 cancer and 50 normal samples in LIHC (B) mRNA expression levels of miR‐383 in four HCC cell lines quantified by RT‐PCR. (C,E) Cell viability of four HCC cell lines under various concentrations of Dox for 48 and 72 h, as assayed by CCK‐8. (D,F) The IC_50_ value of Dox for 48 and 72 h, in the four HCC cell lines calculated according to the CCK‐8 results. Dox, Doxorubicin; HCC, hepatocellular carcinoma; CCK‐8, cell counting kit‐8

To investigate the potential role of miR‐383 in HCC, we first used starBase v.3 project to analyse the level of miR‐383 in LIHC (liver HCC). As expected, the level of miR‐383 was higher in 370 cancer than 50 normal sample in LIHC (Figure 1A). Then, we examined the expression level of miR‐383 in multiple HCC cell lines using RT‐PCR. The results showed that miR‐383 expression was the highest in Huh7, second in HepG2, third in SNU387 and fourth in SNU449 (Figure 1B). MicroRNA‐383 showed higher expression in Huh‐7 compared to that in the normal liver cell line HL‐7702 (Figure 1B). We then detected the cell viability and Dox IC_50_ value of HCC cells treated with different concentrations of Dox. The CCK‐8 assay showed that the cell viability of the HCC cell lines was highest in SNU449 and lowest in Huh7, which was the opposite trend to miR‐383 expression (Figure 1C,E). In addition, the trend of the IC_50_ value of Dox was also opposite to the miR‐383 expression (Figure 1D,F). Thus, these results indicated that miR‐383 expression might correlate with Dox resistance to some extent.

### 3.2 Overexpression of miR‐383 enhances the Dox sensitivity of HCC cells

**Figure 2:**
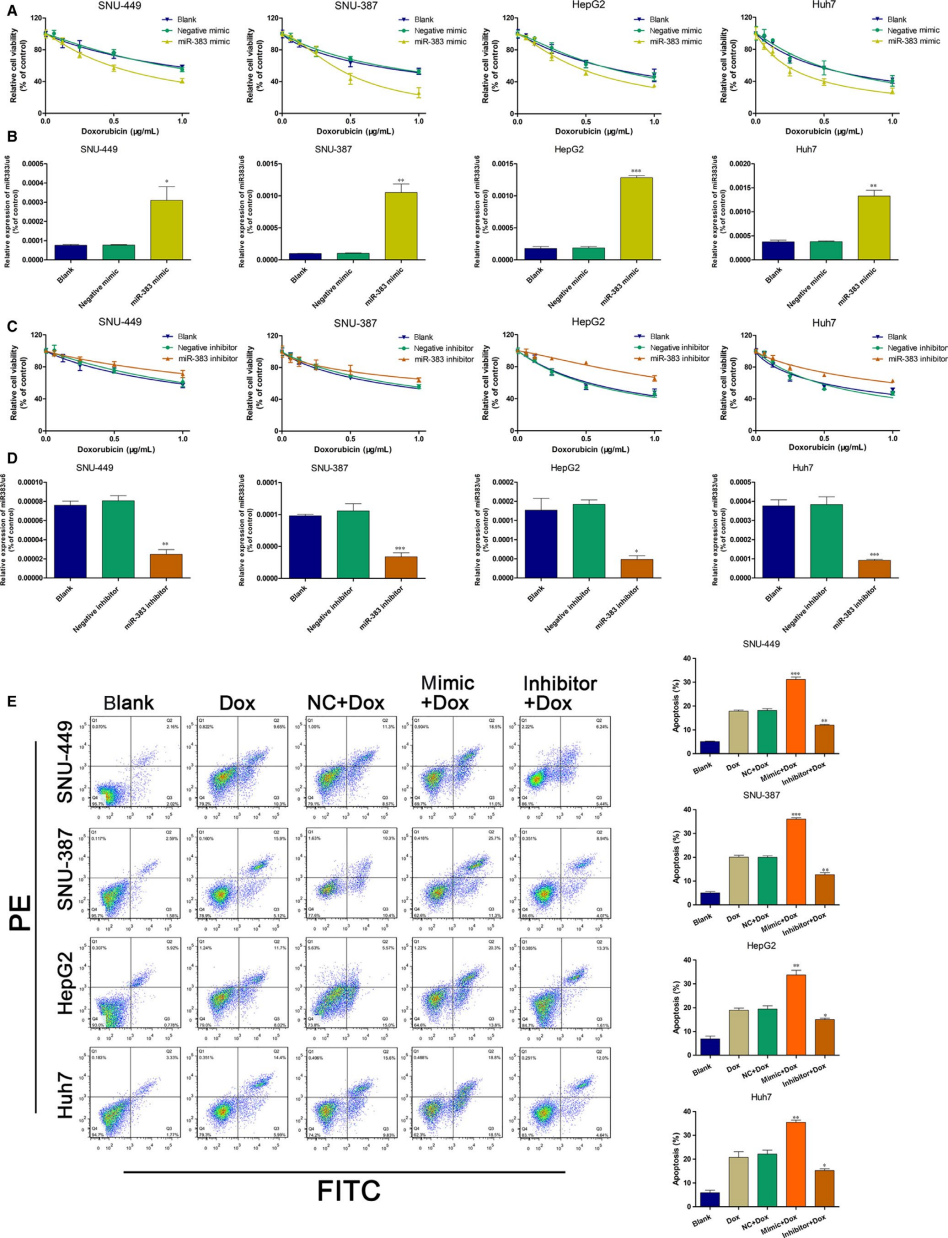
Overexpression of microRNA‐383 (miR‐383) sensitizes HCC cells to Dox. A, The cell viability of four HCC cells lines transfected with miR‐383 mimics, negative control RNA or blank was detected after incubation with at various Dox concentrations (0, 0.5, and 1.0 μg/mL). B, The overexpression efficiency of miR‐383 mimics was detected by RT‐PCR. *P < 0.05, **P < 0.01, ***P < 0.001 versus blank. C, The cell viability of four HCC cells lines transfected with miR‐383 inhibitor, negative control RNA or blank was detected when incubated with various Dox concentrations. D, The inhibition efficiency of miR‐383 inhibitor was detected by RT‐PCR. *P < 0.05, **P < 0.01, ***P < 0.001 versus blank. E, Apoptotic cells were determined using flow cytometry in four HCC cell lines after treatment with blank, 1.0 μg/mL Dox, 1.0 μg/mL Dox + negative control RNA, 1.0 μg/mL Dox + miR‐383 mimics, or 1.0 μg/mL Dox + miR‐383 inhibitor. *P < 0.05, **P < 0.01,***P < 0.001 versus blank. Dox, Doxorubicin; HCC, hepatocellular carcinoma.

To further explore the role of miR‐383 in regulating HCC Dox resistance, we performed gain‐ and loss‐of‐function experiments, using miR‐383 mimics or an inhibitor respectively. We found that overexpression of miR‐383 significantly increased Dox‐induced cytotoxicity (Figure 2A). The overexpression efficiency of the miR‐383 mimics was detected using RT‐PCR (Figure 2B). In contrast, miR‐383 knockdown had the opposite effect (Figure 2C). The knockdown efficiency of miR‐383 inhibitor is shown in Figure 2D. Additionally, we found that overexpression of miR‐383 markedly increased the Dox‐induced cell apoptosis of HCC cells (Figure 2E). These results suggested that miR‐383 restoration promoted the chemosensitivity of HCC cells to Dox.

### 3.3 *EIF5A2* is a direct target of miR‐383

**Figure 3:**
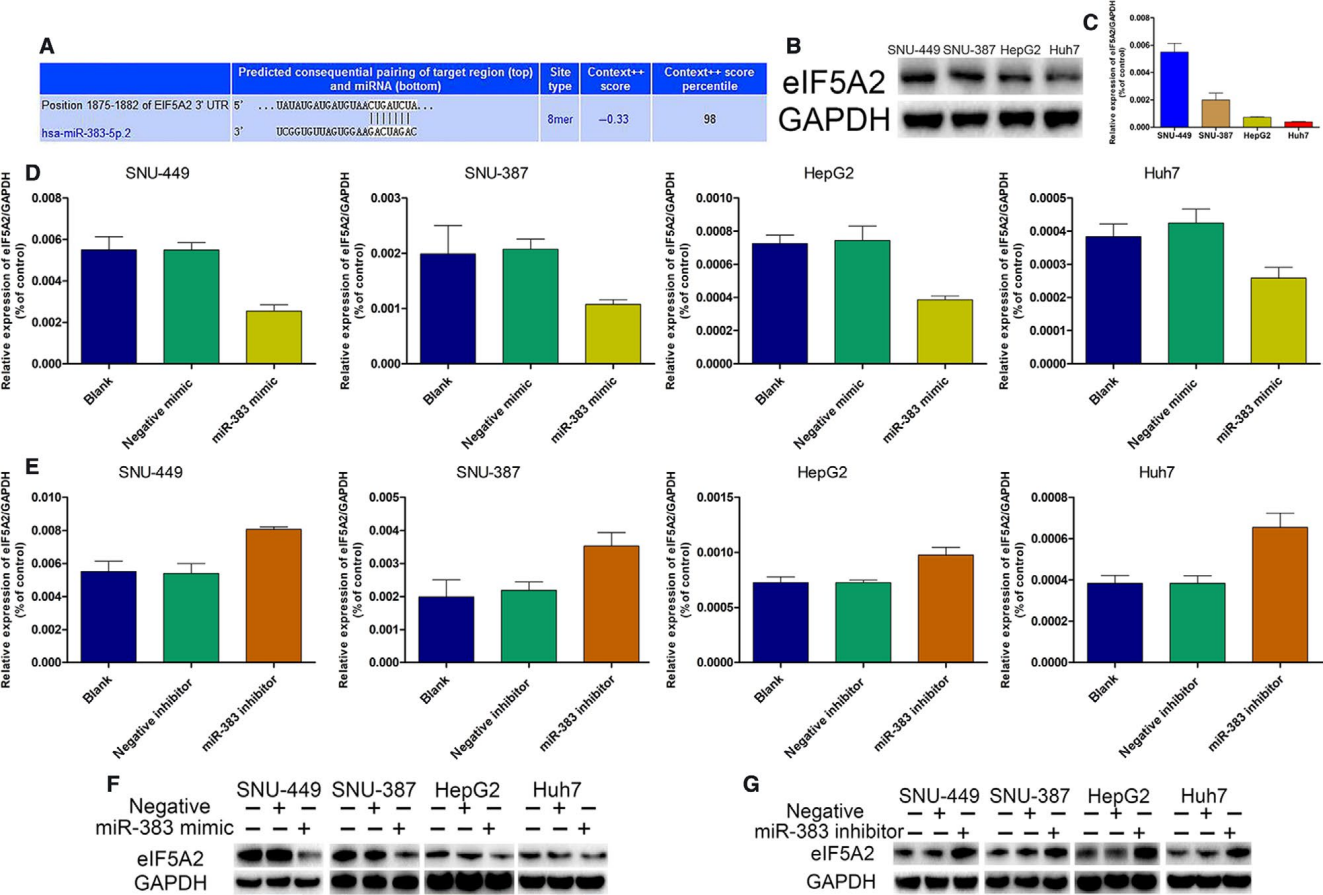
*EIF5A2* acts as a direct target of miR‐383 in HCC cells. A, The bioinformatics software TargetScan was used to identify the binding region of miR‐383 in the *EIF5A2* 3′‐UTR. (B,C) Protein and mRNA expression levels of EIF5A2 in four HCC cell lines quantified by western blotting and RT‐PCR. (D,E) The effect of miR‐383 mimics and inhibitor on the mRNA expression of *EIF5A2* quantified by RT‐PCR. (F,G) The effect of miR‐383 mimics and inhibitor on the protein expression of EIF5A2 quantified by western blotting. 3′‐UTR, 3′‐untranslated region; HCC, hepatocellular carcinoma; EIF5A2, eukaryotic translation initiation factor 5A2

To identify the candidate target genes of miR‐383 that are associated with cancer chemoresistance, we used the miRNA target‐prediction website TargetScan. Interestingly, we found that *EIF5A2*, a vital gene for cancer drug resistance,^18^, ^19^ was predicted as a potential target gene of miR‐383. The 3′ UTR of *EIF5A2* has a binding region for miR‐383 (Figure 3A). Furthermore, we first used starBase v.3 project to analyse the level of *EIF5A2* in LIHC. The results showed that the level of *EIF5A2* was higher in 374 cancer than 50 normal sample in LIHC (Figure S1).To confirm whether *EIF5A2* was the genuine target gene of miR‐383, we first examined the protein and mRNA expression of EIF5A2 in HCC cell lines. The results showed that the expression level of EIF5A2 was highest in SNU449 and lowest in Huh7, which was negatively correlated with miR‐383 expression (Figure 3B,C). We then detected the effect of miR‐383 on EIF5A2 expression using qRT‐PCR and western blotting analysis. The results demonstrated that overexpression of miR‐383 significantly decreased the mRNA and protein expression of EIF5A2 (Figure 3D,F), while miR‐383 knockdown increased EIF5A2 expression (Figure 3E,G). Thus, these results suggested that *EIF5A2* is a target gene of miR‐383.

### 3.4 Knockdown of *EIF5A2* sensitized HCC cells to Dox and reversed the effect of miR‐383 inhibition in regulating Dox resistance

**Figure 4:**
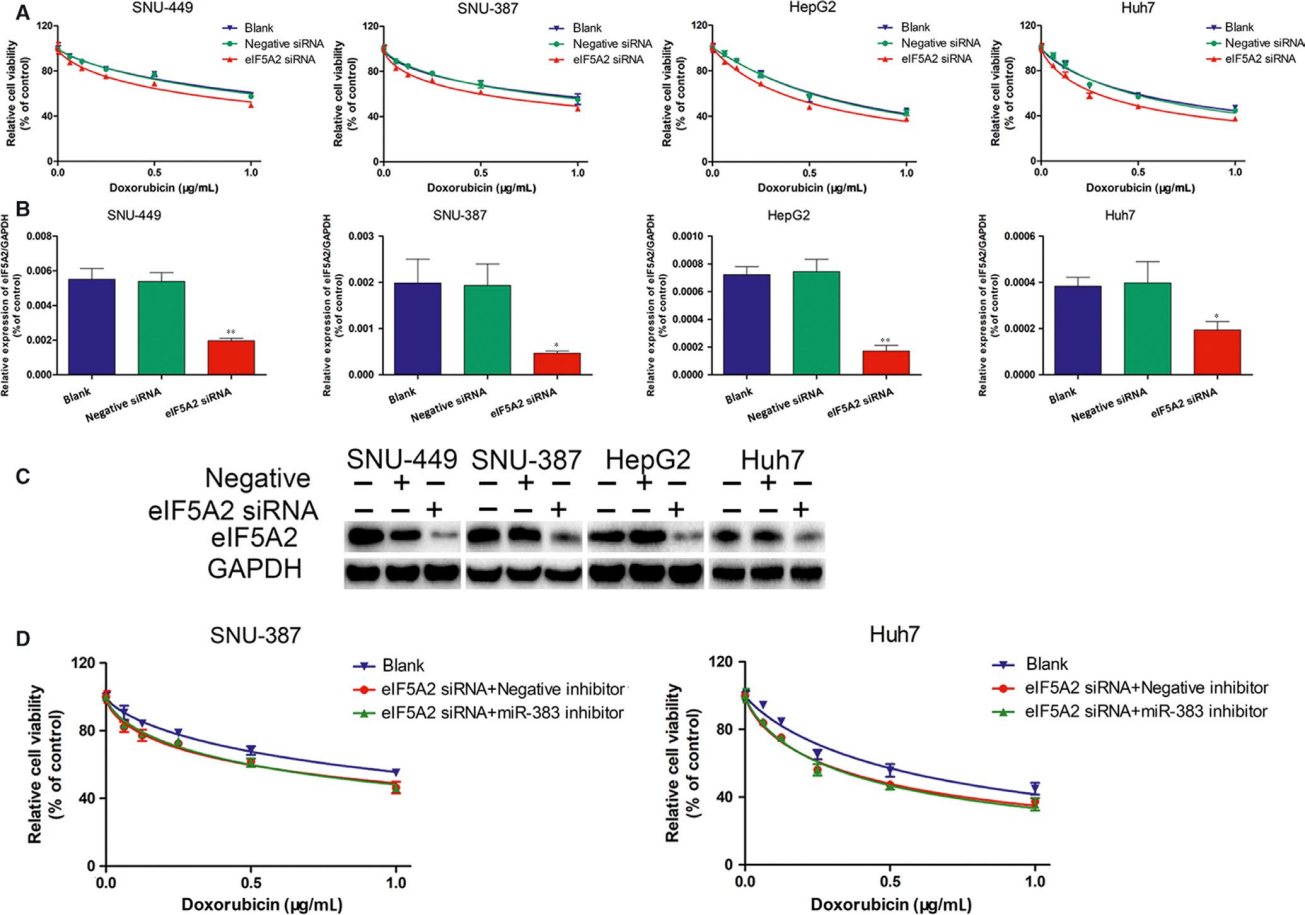
Knockdown of *EIF5A2* sensitizes HCC cells to Dox and reversed the effect of miR‐383 inhibition in regulating Dox resistance. A, The cell viability of four HCC cells lines transfected with the *EIF5A2* siRNA, negative control RNA or blank was detected when incubated with at various Dox concentrations (0, 0.5, 1.0 μg/mL). (B,C) The knockdown efficiency of the *EIF5A2* siRNA was confirmed by RT‐PCR and western blotting. *P < 0.05, **P < 0.01 versus blank. D, The cell viability of four HCC cell lines cultured at various Dox concentrations (0, 0.5 and 1.0 μg/mL) after transfection with blank, *EIF5A2* siRNA + negative control RNA and *EIF5A2* siRNA + miR‐383 inhibitor. Dox, Doxorubicin; HCC, hepatocellular carcinoma; EIF5A2, eukaryotic translation initiation factor 5A2; siRNA, short interfering RNA.

To verify whether miR‐383 regulates Dox resistance by directly targeting *EIF5A2*, we first investigated the role of EIF5A2 in Dox resistance in HCC cells. The results showed that *EIF5A2* knockdown promoted the Dox sensitivity of HCC cells, which was consistent with miR‐383 overexpression (Figure 4A), and the knockdown efficiency of the *EIF5A2* siRNA was confirmed by RT‐PCR and western blotting (Figure 4,C). We then performed rescue experiments by cotransfecting *EIF5A2* siRNA and the miR‐383 inhibitor in HCC cells. We found that miR‐383 inhibition‐mediated Dox resistance was reversed by the *EIF5A2* siRNA (Figure 4D). These findings indicated that miR‐383 alleviated Dox resistance of HCC cells by regulating *EIF5A2* expression.

### 3.5 MiR‐383 overexpression sensitized HCC cells to DOX in vivo

**Figure 5:**
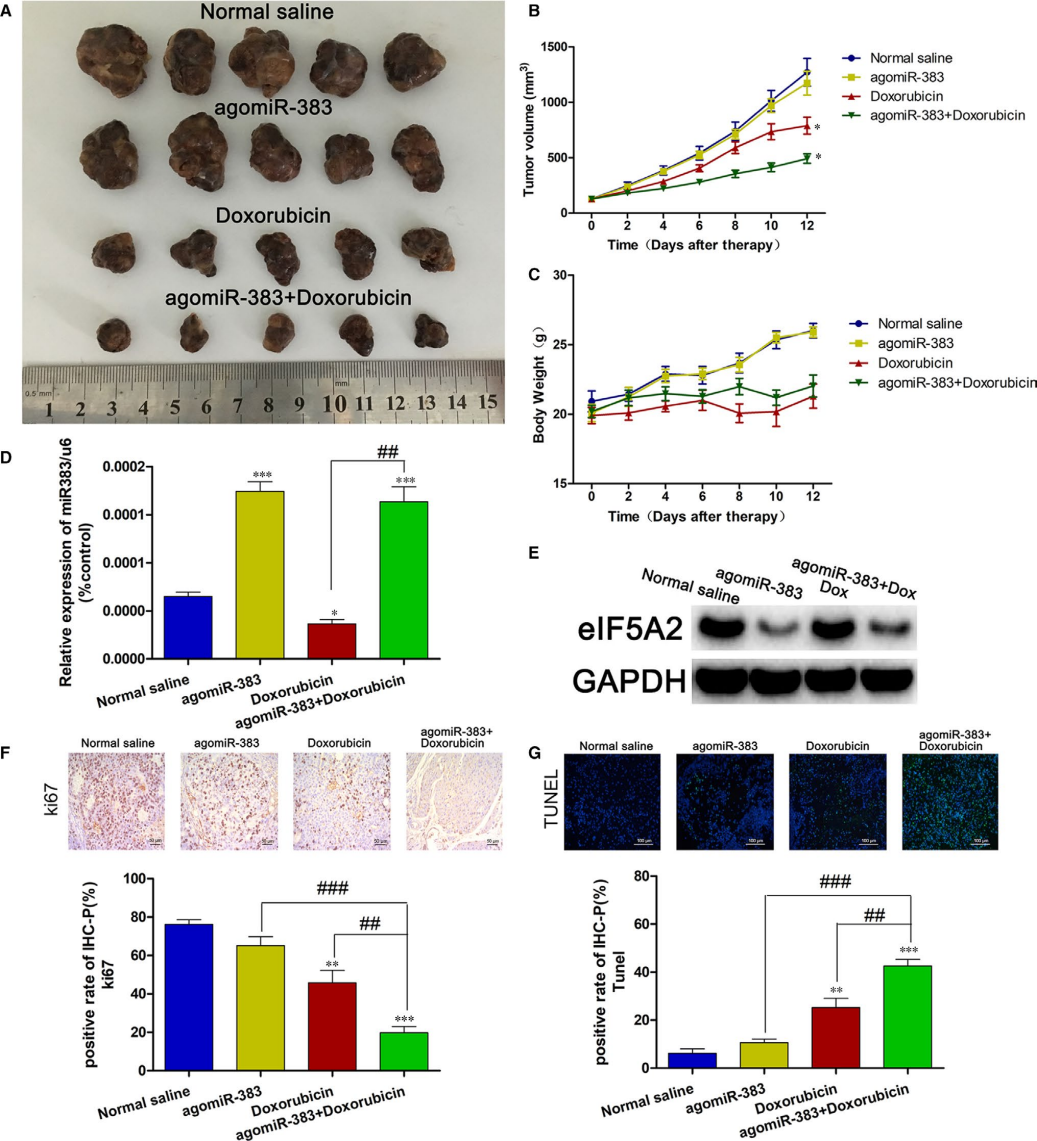
MicroRNA‐383 overexpression sensitizes HCC cells to Dox in vivo. A, Image of the tumours in each group of nude mice treated with normal saline, agomiR‐383, 3 mg/kg Dox or 2 nmol miR‐383 + 3 mg/kg Dox. B, The tumour growth curve. The volume of the tumour was monitored using the following formula: volume = (length × width^2^)/2. C, The bodyweight curve of the nude mice in each group. D, mRNA expression of miR‐383 in the tumour tissues of the nude mice in each group quantified using RT‐PCR. E, Protein expression of EIF5A2 in the tumour tissues of nude mice in each group quantified by western blotting. F, The marker of proliferation Ki‐67 (ki‐67) staining in the tumour tissues of the nude mice in each group. **P < 0.01, ***P < 0.001 versus Normal saline. ^##^ *P* < 0.01, ^###^ *P* < 0.001. G, TUNEL assay was used to detected apoptosis in the tumour tissues of the nude mice in each group. **P < 0.01, ***P < 0.001 versus Normal saline. ^##^ *P* < 0.01, ^###^ *P* < 0.001. Dox, Doxorubicin; HCC, hepatocellular carcinoma; EIF5A2, eukaryotic translation initiation factor 5A2; TUNEL, terminal deoxynucleotidyl transferase dUTP nick end labelling.

To further investigate the role of miR‐383 in Dox resistance in HCC cells, we employed a subcutaneous xenotransplanted nude mouse model to detect the role of miR‐383 in vivo. The results showed that injection of both agomiR‐383 and Dox into a tumour could significantly inhibit tumour growth compared to injection of agomiR‐383 alone or Dox alone (Figure 5A). In addition, the tumour growth curve and nude mice bodyweight curve confirmed this result (Figure 5B,C). The injection of agomiR‐383 was successful and the miR‐383 level was up‐regulated in the tumour tissues (Figure 5D). Meanwhile, we examined the expression of *EIF5A2* in the nude mice tumour tissues, the results demonstrated that miR‐383 could inhibit EIF5A2 expression in vivo (Figure 5E). Finally, marker of proliferation Ki‐67 (ki‐67) staining and TUNEL assays indicated that miR‐383 overexpression could enhance growth inhibition and promote apoptosis of HCC cells in vivo (Figure 5F,G).

## 4 DISCUSSION

Studies have shown that for patients with advanced HCC, resistance to systemic chemotherapy and TACE have been the main obstacles to longer survival.^20^, ^21^ Therefore, there is an urgent need to thoroughly explore the molecular mechanisms of chemoresistance in HCC to develop better strategies for prolonging the survival of patients with HCC. Recently, miRNAs have been proven to play a vital role in regulating the chemoresistance and progression of cancers. In HCC, accumulating evidence has demonstrated that dysregulated miRNAs correlate with HCC chemoresistance.^22^, ^23^ However, the roles of miRNAs are complicated because one miRNA may have multiple target genes. Thus, more studies are needed to clarify the functions of certain miRNAs in cancers.

It was reported that miR‐383 is frequently down‐regulated and acts as a tumour suppressor in multiple cancers.^24^ In ovarian cancer, the expression of miR‐383 was proved to be significantly down‐regulated in ovarian cancer tissues and ovarian cancer cell lines, and miR‐383 regulated *LDHA* expression in the ovarian cancer cells to inhibit glycolysis, cell proliferation and invasion.^25^In colorectal cancer, miR‐383 was reported to act as a tumour suppressor by modulating *CREPT* (encoding cell‐cycle related and expression‐elevated protein in tumour, also known as RPRD1B) expression.^26^ In lung cancer, miR‐383 could suppress lung cancer progression by targeting *EPAS1* (encoding endothelial PAS domain‐containing protein 1).^13^ In HCC, only two studies have stated a role of miR‐383.^15^, ^16^ However, there is no evidence of the role of miR‐383 in HCC chemoresistance. In the present study, we observed that the expression level of miR‐383 correlated negatively with Dox sensitivity. Overexpression of miR‐383 sensitized HCC cells to Dox‐induced cytotoxicity and apoptosis, whereas miR‐383 knockdown had the opposite effects.

Eukaryotic translation initiation factor 5A2 (EIF5A2), one isoform of EIF5A, plays a vital role in mRNA translation.^27^ Emerging data show that EIF5A2 acts as an oncogene and plays a vital role in regulating chemoresistance in human cancers.^28^, ^29^, ^30^, ^31^, ^32^ In colon cancer, EIF5A2 was reported to enhance chemoresistance to Dox via regulation of the epithelial‐mesenchyme transition,^33^ and in oesophageal squamous cell carcinoma cells, EIF5A2 overexpression led to chemoresistance to 5‐fluorouracil (5‐FU), docetaxel and taxol.^18^ In HCC, EIF5A2 was proven to be a target of N1‐Guanyl‐1,7‐Diaminoheptane (GC7, a deoxyhypusine synthase inhibitor), and inhibition of EIF5A2 by GC7 could sensitize HCC cells to Dox.^34^, ^35^ EIF5A2 was reported to be regulated by miR‐125b, miR‐29b and miR‐9^36^, ^37^, ^38^ in HCC. However, chemoresistance and progression of HCC are very complicated. Multiple miRNAs and their target genes are likely to be involved in HCC. Therefore, further studies are needed to clarify the potential molecular mechanism of HCC. In the present study, we proved that *EIF5A2* is a target gene of miR‐383 and is involved in Dox resistance.

In summary, the present study explored the relationship between the expression of miR‐383 and *EIF5A2* and Dox resistance in HCC and validated that *EIF5A2* is indeed a target gene of miR‐383. MiR‐383 overexpression could sensitize HCC cells to Dox via targeting *EIF5A2*. Our study proved the function of the miR‐383‐*EIF5A2* axis in chemoresistance of HCC. Targeting *EIF5A2* via miR‐383 might be a novel and effective therapeutic strategy to relieve chemoresistance in HCC.

## CONFLICT OF INTEREST

The authors declare that they have no competing interests.
