# Supplementary figures and images for: MicroRNA‐383 inhibits doxorubicin resistance in hepatocellular carcinoma by targeting eukaryotic translation initiation factor 5A2
Source: J Cell Mol Med. 2019 Feb 23;23(11):7190–9. doi: 10.1111/jcmm.14197 (PMC6815770; doi:10.1111/jcmm.14197)

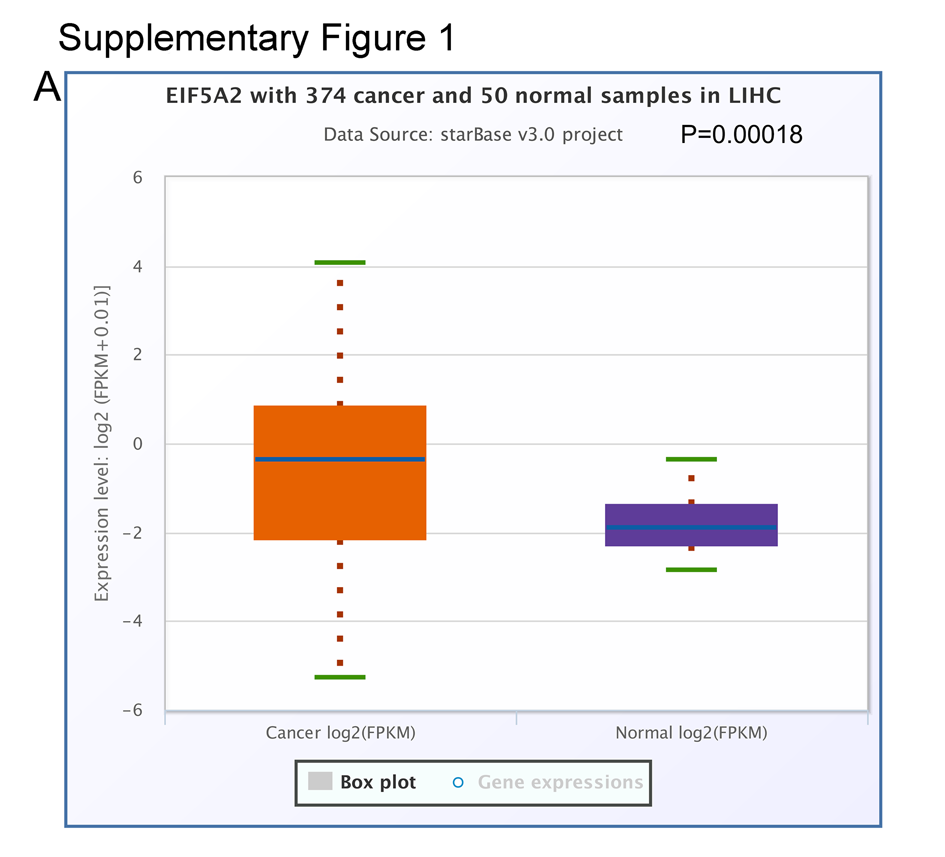

Supplement: Supplementary file 1 [file JCMM-23-7190-s001.tif]
