# Supplementary material for: MicroRNA‐383 inhibits doxorubicin resistance in hepatocellular carcinoma by targeting eukaryotic translation initiation factor 5A2
Source: J Cell Mol Med. 2019 Feb 23;23(11):7190–9. doi: 10.1111/jcmm.14197 (PMC6815770; doi:10.1111/jcmm.14197)
Supplement: Supplementary file 2 [file JCMM-23-7190-s002.docx]

**Figure S1** We used starBase v 3.0 project to analyze the expression of EIF5A2 in 374 cancer and 50 normal samples in LIHC
